# A Prognostic Prediction Model Developed Based on Four CpG Sites and Weighted Correlation Network Analysis Identified *DNAJB1* as a Novel Biomarker for Pancreatic Cancer

**DOI:** 10.3389/fonc.2020.01716

**Published:** 2020-08-25

**Authors:** Lingming Kong, Peng Liu, Xiang Fei, Tianyu Wu, Zhongpeng Wang, Baohui Zhang, Jiatong Li, Xiaodong Tan

**Affiliations:** ^1^Department of General Surgery, Shengjing Hospital of China Medical University, Shenyang, China; ^2^Department of Cardiology, The First Affiliated Hospital of China Medical University, Shenyang, China; ^3^Department of Physiology, School of Life Sciences, China Medical University, Shenyang, China; ^4^Department of Orthopedics, The First Affiliated Hospital of China Medical University, Shenyang, China

**Keywords:** LASSO, WGCNA, pancreatic cancer, DNA methylation, prognostic prediction, DNAJB1

## Abstract

**Background:**

The prognosis of pancreatic cancer, which is among the solid tumors associated with high mortality, is poor. There is a need to improve the overall survival rate of patients with pancreatic cancer.

**Materials and Methods:**

The Cancer Genome Atlas (TCGA) dataset with 153 samples and the International Cancer Genome Consortium (ICGC) dataset with 235 samples were used as the discovery and validation cohorts, respectively. The least absolute shrinkage and selection operator regression was used to construct the prognostic prediction model based on the DNA methylation markers. The predictive efficiency of the model was evaluated based on the calibration curve, concordance index, receiver operating characteristic curve, area under the curve, and decision curve. The xenograft model and cellular functional experiments were used to investigate the potential role of *DNAJB1* in pancreatic cancer.

**Results:**

A prognostic prediction model based on four CpG sites (cg00609645, cg13512069, cg23811464, and cg03502002) was developed using TCGA dataset. The model effectively predicted the overall survival rate of patients with pancreatic cancer, which was verified in the ICGC dataset. Next, a nomogram model based on the independent prognostic factors was constructed to predict the overall survival rate of patients with pancreatic cancer. The nomogram model had a higher predictive value than TCGA or ICGC datasets. The low-risk group with improved prognosis exhibited less mutational frequency and high immune infiltration. The brown module with 247 genes derived from the WGCNA analysis was significantly correlated with the prognostic prediction model, tumor grade, clinical stage, and T stage. The bioinformatic analysis indicated that *DNAJB1* can serve as a novel biomarker for pancreatic cancer. *DNAJB1* knockdown significantly inhibited the proliferation, migration, and invasion of pancreatic cancer cells *in vivo* and *in vitro*.

**Conclusion:**

The prognostic prediction model based on four CpG sites is a new method for predicting the prognosis of patients with pancreatic cancer. The molecular characteristic analyses, including Gene Ontology, Gene Set Enrichment Analysis, mutation spectrum, and immune infiltration of the subgroups, stratified by the model provided novel insights into the initiation and development of pancreatic cancer. *DNAJB1* may serve as diagnostic and prognostic biomarkers for pancreatic cancer.

## Introduction

Pancreatic cancer, which is one of the gastrointestinal tract malignancies associated with high mortality, is the fourth most common cause of cancer-related deaths in the United States of America (1). Due to the specific anatomical position and malignant phenotype of pancreatic cancer, most patients exhibit insidious onset and unspecific clinical symptoms at the earlier stage of pancreatic cancer. Large proportions of patients with pancreatic cancer are diagnosed at an advanced stage along with early distant metastasis and neural or vascular invasion. Thus, patients with pancreatic cancer exhibit a low survival rate with a 5-year survival rate of less than 5% (2, 3). Currently, the classical TNM staging and blood tumor markers (CA 19–9, CA 125, and CEA) are used to assess the risk level in patients with pancreatic cancer and predict the prognosis, which are not highly efficient or accurate (4, 5). There is an urgent need to devise strategies to increase the overall survival rate of patients with pancreatic cancer, which can be achieved by developing a sensitive and specific risk prediction model for prognosis. The novel biomarkers derived from the risk prediction model can serve as diagnostic, therapeutic, and prognostic biomarkers for pancreatic cancer.

The initiation and progression of various cancers are reported to be regulated by epigenetic alterations. DNA methylation, an important epigenetic regulation, silences tumor suppressor genes, and upregulates oncogenic genes through hypermethylation and hypomethylation of the corresponding CpG islands in the promoter regions, respectively. Several studies have demonstrated that numerous genes with deregulated methylation status, such as *KRAS*, *CDKN2A*, *TP53*, *CD1D*, *MUC4*, and *MUC1* play vital roles in the progression of pancreatic cancer (6–8). Moreover, specific DNA methylation signatures in the circulating DNA from pancreatic juice and plasma can be used as novel biomarkers for pancreatic cancer (9, 10). Several prognostic prediction models using DNA methylation data have been proposed for prostate, gastric, colorectal, and esophageal cancers (11–14). These studies indicated that the DNA methylation status is closely associated with the prognosis of multiple cancers. The development of high-throughput sequencing and construction of large cancer genome databases, such as The Cancer Genome Atlas (TCGA) and International Cancer Genome Consortium (ICGC) have enabled access to massive sequencing data and the corresponding clinical data. This study aimed to explore the potential prognostic values of prognostic prediction model generated based on CpG sites in pancreatic cancer.

In this study, a prognostic prediction model based on four CpG sites was established using TCGA dataset. The conclusion of this model was verified in the external ICGC dataset. Next, this study demonstrated that the nomogram model generated using the independent prognostic factors can be used as an efficient tool for prognostic prediction. Additionally, the comparisons between molecular subgroups based on the prognostic prediction model identified novel biomarkers and therapeutic targets for pancreatic cancer. The molecular characteristic analyses among subgroups may aid in elucidating the mechanisms underlying pancreatic cancer.

## Materials and Methods

### Downloading and Preprocessing Data

The DNA methylation, RNA sequencing (RNA-Seq; HTSeq counts type), single nucleotide variation (MuTect type) data of patients with pancreatic cancer were downloaded from TCGA database^1^. The latest clinicopathological information and clinical follow-up data of patients with pancreatic cancer in TCGA were downloaded on 13 November 2019 (15). The DNA methylation status and clinical data of patients with pancreatic cancer in the ICGC database (the Australian Pancreatic Cancer Genome Initiative, https://icgc.org) were used as the validation cohort (16). In the DNA methylation data, the CpG sites with absent values in >70% of the samples were removed and the K-nearest neighbor algorithm was used to estimate and replace the missing values. The probes from upstream 2 kb to downstream 200 bp of the transcription start site region were used for further analysis. In the RNA-Seq data, the genes with missing values in >50% of the total samples were deleted. The silent mutation and mutation in the intron region of single nucleotide variation data were deleted (17). The detailed information of the pancreatic cancer samples obtained from TCGA and ICGC databases is shown in Supplementary Tables 1, 2, respectively.

### Construction of the Prognostic Prediction Model Based on CpG Sites

The Cancer Genome Atlas dataset was used as the discovery cohort. The differentially methylated CpG sites between 10 normal and 185 tumor samples were identified from TCGA database. The R package “minfi” was used to normalize the β-values of methylation data. The Mann–Whitney *U* test was performed to select the differentially methylated CpG sites with adjusted *p*-value < 0.05 and | log2 fold-change| > 2 (11, 18). The samples of patients with pancreatic cancer exhibiting survival time of less than 30 days were removed. In total, 153 samples were selected to identify the survival-related CpG sites. The CpG sites with *p*-value < 0.05 in both Cox and log-rank tests were used for the generation of prognostic prediction model. The least absolute shrinkage and selection operator (LASSO) regression was used to construct the prognostic prediction model using the R package “glmnet” (19, 20). To verify the effectiveness of the model, the ICGC dataset with 235 pancreatic cancer samples was used as the validation cohort.

### Nomogram Model Development

To comprehensively utilize the clinicopathological data to increase the predictive ability of the LASSO model, the independent prognostic factors were identified based on the univariate and multivariate Cox analyses. The nomogram was generated based on the selected independent prognostic factors and used to predict the 1-, 3-, and 5-year overall survival rates of patients with pancreatic cancer. The discriminative ability of the nomogram model was evaluated based on the calibration curve, concordance index (C-index), receiver operator characteristic (ROC) curve, and area under the curve (AUC). The decision curve analysis (DCA) was used to compare the clinical benefits among these models. The R packages “rms,” “survcomp,” “timeROC,” “survival,” and “stdca.R” were used for the analysis (21–23).

### Molecular Characteristic Analyses of the Prognostic Prediction Model

To further explore the mechanisms underlying the prognostic prediction model, several molecular characteristic analyses were performed using the high-risk and low-risk groups depending on the model. The immunological infiltrations of six types of immune cells were calculated using TIMER (Tumor Immune Estimation Resource, https://cistrome.shinyapps.io/timer/) (24, 25). The R package “maftools” was used to perform the mutation spectrum analysis (26). The R packages “clusterProfiler” and “ggplot2” were utilized to perform and visualize the results of Gene Ontology (GO) analysis and Gene Set Enrichment Analysis (GSEA) (27, 28).

### Weighted Correlation Network Analysis (WGCNA) to Identify the Hub Genes Associated With the Model

The prognostic prediction model effectively predicted the prognosis of the patients with pancreatic cancer and categorized the samples into high-risk and low-risk groups. The differentially expressed genes between the high-risk and low-risk groups may play a vital role in the progression of pancreatic cancer. The differentially expressed genes between different groups were calculated and selected using the R package “DESeq2” with an adjusted *p*-value < 0.05 and | log2 fold-change| > 1 (29). To identify the most relevant genes of the model, weighted correlation network analysis (WGCNA) was performed according to the official guideline of the R package “WGCNA” (30, 31). The parameters used in the analysis were set as follows: best soft power threshold, 4; minimum module size, 30; merge cut height, 0.25. The Cytoscape software (version 2.8.3) was used to calculate and visualize the hub genes in the gene network (32, 33).

### Gene Expression Profiling Interactive Analysis (GEPIA), Kaplan-Meier (KM) Plotter, and TISIDB Databases

The Gene expression profiling interactive analysis (GEPIA) website provided the differentially expressed genes between 179 pancreatic tumor samples and 171 normal samples based on the integrated RNA-Seq data from TCGA and Genotype-Tissue Expression (GTEx) databases (34). The KM plotter website provided the genes associated with overall survival and relapse-free survival of patients from TCGA dataset (35). The TISIDB website was used to analyze the relationship between clinicopathological information and gene expression (36).

### Cell Culture and Transfection

The four human pancreatic cancer cell lines (AsPC-1, Capan-2, MIA PaCa-2, and SW1990) and one human normal pancreatic cell line (hTERT-HPNE) used in this study were purchased from the American Type Culture Collection (ATCC). The cells were cultured following the official guidelines provided in the ATCC website at 37°C and 5% CO_2_. The pHBLV-U6-ZsGreen-puro lentiviral RNAi expression system containing the *DNAJB1* shRNA sequence (5′-GGTGCCAATGGTACCTCTTTC-3′) were designed and provided by Hanbio Biotechnology Co. Ltd. (Shanghai, China).

### Western Blotting and Immunohistochemical Assay

The western blotting analysis was performed following the methods of a previous study (37). Equal amounts (30 μg) of protein were subjected to sodium dodecyl sulfate-polyacrylamide gel electrophoresis (SDS-PAGE) using a 10% gel. The following primary antibodies used for the western blotting analysis were purchased from Proteintech Group (Rosemont, United States): anti-DNAJB1 (Catalog number: 13174-1-AP; 1:1000): and anti-alpha tubulin (Catalog number: 11224-1-AP; 1:3000) antibodies. The immunohistochemical assay was performed following the methods of a previous study (38).

### Cell Proliferation, Invasion, and Migration Assays

The CCK-8 and colony formation assays were used to estimate the proliferative ability of different groups. For CCK-8 assay, 2000 cells of different groups were seeded into a 96-well plate. The cells in each well were incubated with 10 μL of CCK-8 solution (Beyotime biotechnology Co. Ltd., Shanghai, China) for 60 min. The optical density of the mixture was measured at 450 mm using a microplate spectrophotometer. The colony formation assay was performed using 1000 cells of different groups seeded in a 6-well plate. The culture medium was replaced every 3 days. After the appearance of visible colonies, 4% paraformaldehyde and crystal violet were used to fix and stain the colonies. The transwell and wound healing assays were used to analyze the cellular invasion and cellular migration, respectively. These assays were performed following the methods described in a previous study (37). The 96-well plates, 6-well plates, transwell system, and cell culture flask were purchased from Guangzhou Jet Bio-Filtration Co., Ltd.

### Xenograft Tumor Mouse Model

The subcutaneous tumor mouse model was used to assess the tumor cell proliferative ability *in vivo* following the method described in a previous study (39). Twelve BALB/c nude mice (4-week-old, female) were purchased from Huafukang Biotechnology Co. Ltd. (Beijing, China). The cells (1 × 10^6^) of different groups in 100 μL phosphate buffer solution were injected into the right axillary area of each nude mouse. The subcutaneous tumor volume was measured and recorded once a week. The tumor volume was measured as follows: volume = 0.5 × L × W^2^, where L is the long axis of the tumor and W is the short axis of the tumor.

### Statistical Analysis

The statistical analyses were performed in the R software (version 3.5.3) and RStudio software. The data were analyzed by two-tailed Student’s *t*-test and one-way analysis of variance (ANOVA). The difference was considered statistically significant when the *p*-value was less than 0.05.

## Results

### Construction of the Prognostic Prediction Model Based on Four CpG Sites

**FIGURE 1:**
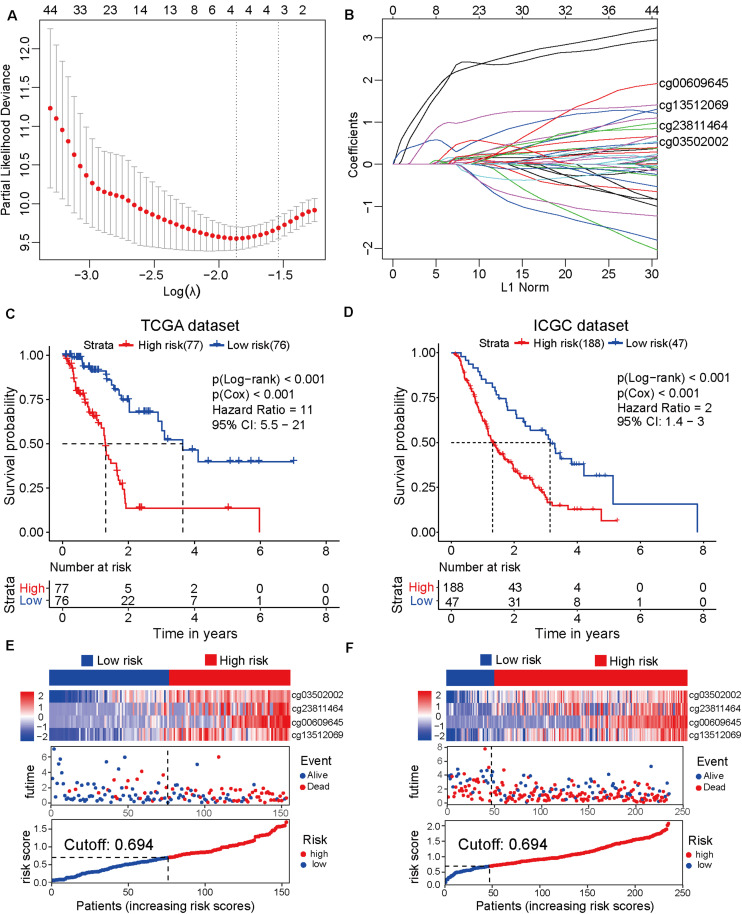
Development and verification of the prognostic prediction model based on four CpG sites by least absolute shrinkage and selection operator (LASSO) regression. **(A)** The selection of tuning parameter (λ) in the LASSO model based on the 10–fold cross–validation with minimum criteria. The log(λ) value of -1.863678 is used for further analysis. **(B)** The four CpG sites (cg00609645, cg13512069, cg23811464, and cg03502002) and their coefficients were used to construct the model. **(C)** The Cancer Genome Atlas (TCGA) dataset (discovery cohort) is divided into high-risk (*N* = 77) and low-risk (*N* = 76) groups based on the risk scores generated from the LASSO model. The Kaplan-Meier survival plots of high-risk and low-risk groups. **(D)** The Kaplan-Meier survival plot of the high-risk (*N* = 188) and low-risk (*N* = 47) groups of the International Cancer Genome Consortium (ICGC) dataset (validation cohort). **(E)** From top to bottom, the heatmap of the four CpG sites in the high-risk and low-risk groups of TCGA dataset (top). The distribution plot of survival time and survival status of high-risk and low-risk groups of TCGA dataset (middle). The *X*-axis is the patients’ number with increasing risk scores and the *Y*-axis is the survival time. The distribution plot of the risk scores of the high-risk and low-risk groups of TCGA dataset (bottom). **(F)** The heatmap of the four CpG sites of the high-risk and low-risk groups of the ICGC dataset (top). The distribution plot of survival time and survival status of the high-risk and low-risk groups of the ICGC dataset (middle). The distribution plot of the risk scores of the high-risk and low-risk groups of the ICGC dataset (bottom).

To establish the prognostic prediction model based on CpG sites, 3173 differentially methylated CpG sites were identified among 10 normal and 185 tumor samples from TCGA dataset (Supplementary Table 3). Next, 1325 prognosis-related CpG sites with *p*-value < 0.05 in both Cox and log-rank tests were selected for further LASSO regression analysis (Supplementary Table 4). After the LASSO regression analysis, a prognostic model based on four CpG sites, namely cg00609645, cg13512069, cg23811464, and cg03502002, was developed (Figures 1A,B). The detailed information on the four CpG sites is shown in Supplementary Table 5. Based on the four CpG site β values and the corresponding risk coefficients, each patient was assigned a risk score according to the following formula: risk score = (cg00609645 × 1.461) + (cg13512069×1.226) + (cg23811 464 × 0.539) + (cg03502002 × 0.519). As shown in Figures 1C–E, the samples from TCGA dataset were separated into high-risk and low-risk groups based on the median of the risk scores (cutoff value: 0.694). In order to improve the universality of the prognostic model, the same cutoff value was used in the ICGC dataset. The analysis revealed that the risk score was significantly associated with the overall survival of patients with pancreatic cancer [Hazard ratio (HR), 11; 95% confidence interval (CI), 5.5–21; *p* < 0.001] in the TCGA discovery dataset. Similarly, the risk score also significantly predicted the overall survival of patients with pancreatic cancer (HR, 2; 95% CI: 1.4–3; *p* < 0.001) in the ICGC validation cohort (Figures 1D–F). These results suggested that the prediction model based on four CpG sites can be an effective tool to predict the prognosis of patients with pancreatic cancer.

### Construction of Nomogram Model Based on the Independent Prognosis-Related Factors

**FIGURE 2:**
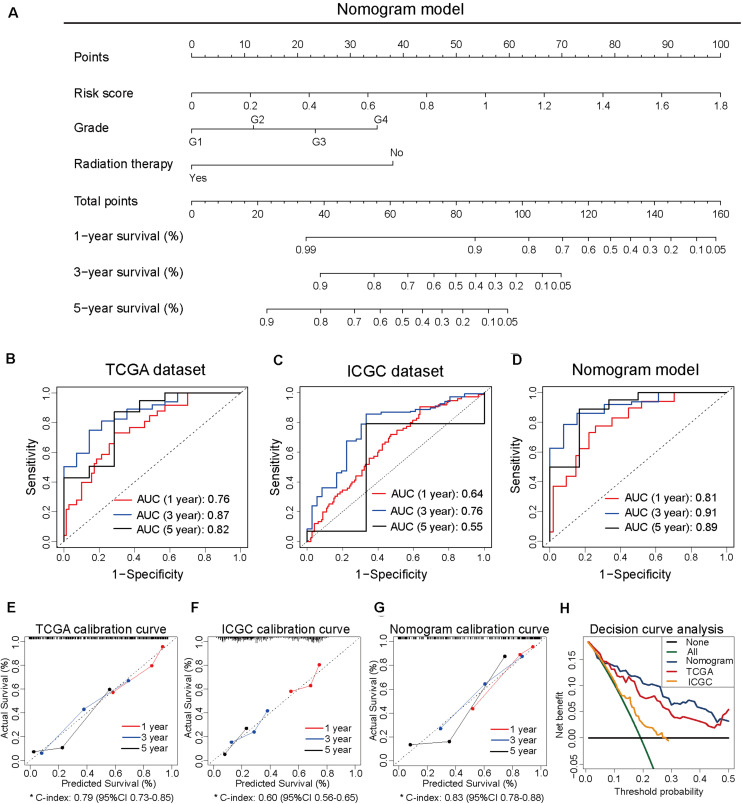
Construction of the nomogram model based on three independent prognosis-related factors. **(A)** The nomogram model developed to predict 1-, 3-, and 5-year survival rates of patients with pancreatic cancer from The Cancer Genome Atlas dataset. **(B)** The receiver operating characteristic (ROC) curves of TCGA dataset to predict the 1-, 3-, and 5-year survival rates. **(C)** The ROC curves of the International Cancer Genome Consortium (ICGC) dataset to predict the 1-, 3-, and 5-year survival rates. **(D)** The ROC curves of the nomogram model to predict the 1-, 3-, and 5-year survival rates. **(E)** The calibration curves for predicting the 1-, 3-, and 5-year survival rates of patients from TCGA dataset. **(F)** The calibration curves for predicting the 1-, 3-, and 5-year survival rates of patients from ICGC dataset. **(G)** The calibration curves for predicting the 1-, 3-, and 5-year survival rates in the nomogram model. **(H)** Decision curve analysis of TCGA, ICGC, and nomogram models.

To develop the nomogram model for predicting the prognosis of patients with pancreatic cancer, the univariate and multivariate Cox analyses were performed using the risk score and other clinicopathological factors. The univariate Cox analysis based on TCGA dataset revealed that the risk score (HR, 10.72; 95% CI, 5.52–20.80; and *p* < 0.001), age (HR, 1.03; 95% CI, 1.01–1.05; and *p* = 0.016), tumor grade (HR, 1.80; 95% CI, 1.29–2.50; and *p* < 0.001), clinical stage (HR, 1.54; 95% CI, 1.03–2.29; and *p* = 0.035), T stage (HR, 2.26; 95% CI, 1.28–4.00; and *p* = 0.005), N stage (HR, 2.47; 95% CI, 1.31–4.66; and *p* = 0.005), site of resection (HR, 0.50; 95% CI, 0.27–0.92; and *p* = 0.026), and radiation therapy (HR, 0.21; 95% CI; 0.07–0.68; and *p* = 0.009) can serve as prognosis-associated factors. According to the general rule, the multivariate Cox analysis was performed using these prognosis-associated factors to avoid the overfitting of the multivariable Cox model. The multivariate Cox analysis revealed that the risk score (HR, 24.68; 95% CI, 7.70–79.14; and *p* < 0.001), tumor grade (HR, 2.33; 95% CI, 1.16–4.65; and *p* = 0.017), and radiation therapy (HR, 0.14; 95% CI 0.04–0.50; and *p* = 0.003) were independent prognosis-related factors (Table 1). The nomogram model was constructed using these independent prognosis factors to predict the 1-, 3-, and 5-year survival rates of patients with pancreatic cancer (Figure 2A).

**TABLE 1:** Univariate and multivariate Cox analyses of clinicopathological information and risk score of the prognostic prediction model.

Prognostic factors	Univariate Cox regression			Multivariate Cox regression		
	HR	95% CI	*P*-value	HR	95% CI	*P*-value
Risk score	10.72	5.52–20.80	< 0.001	24.68	7.70–79.14	< 0.001
Age	1.03	1.01–1.05	0.016	1.00	0.96–1.04	0.977
Gender	0.82	0.49–1.35	0.428			
Grade	1.80	1.29–2.50	< 0.001	2.33	1.16–4.65	0.017
Stage (AJCC 7th)	1.54	1.03–2.29	0.035	0.87	0.23–3.23	0.836
T	2.26	1.28–4.00	0.005	1.80	0.51–6.31	0.360
M	0.55	0.07–4.08	0.560			
N	2.47	1.31–4.66	0.005	1.50	0.59–3.78	0.395
Alcohol history	1.28	0.72–2.26	0.402			
Alcoholic exposure	0.88	0.70–1.10	0.266			
Site of resection	0.50	0.27–0.92	0.026	0.48	0.22–1.05	0.066
Radiation therapy	0.21	0.07–0.68	0.009	0.14	0.04–0.50	0.003
Smoking history	0.90	0.75–1.08	0.273			
Histologic grading	0.71	0.35–1.45	0.346			

To compare the predictive efficiency among the nomogram model, TCGA dataset, and ICGC dataset, the AUC of ROC curve was used to assess the discriminative ability. The nomogram model (1-year: 0.81, 3-year: 0.91, and 5-year: 0.89) exhibited better performance in predicting the survival rates than TCGA dataset (1-year: 0.76, 3-year: 0.87, and 5-year: 0.82) and ICGC dataset (1-year: 0.64, 3-year: 0.76, and 5-year: 0.55; Figures 2B–D). The calibration curve of the three models exhibited satisfactory consistency between the predicted survival rate and the actual survival rate. However, the C-index of the nomogram model (C-index, 0.83; 95% CI, 0.78–0.88) was higher than that of TCGA dataset (C-index, 0.79; 95% CI, 0.73–0.85) and ICGC dataset (C-index, 0.60; 95% CI, 0.56–0.65; Figures 2E–G). Moreover, the DCA curve revealed that the predicted clinical benefits of the nomogram model were better than those of TCGA dataset and ICGC dataset (Figure 2H). These results suggested that the prognostic prediction model based on four CpG sites can serve as an effective model for predicting prognosis in patients with pancreatic cancer. The nomogram model based on the risk score and other independent factors improved the efficiency of the prediction model based on four CpG sites.

### Molecular Characteristics of the Subgroups Based on the Prognostic Prediction Model

**FIGURE 3:**
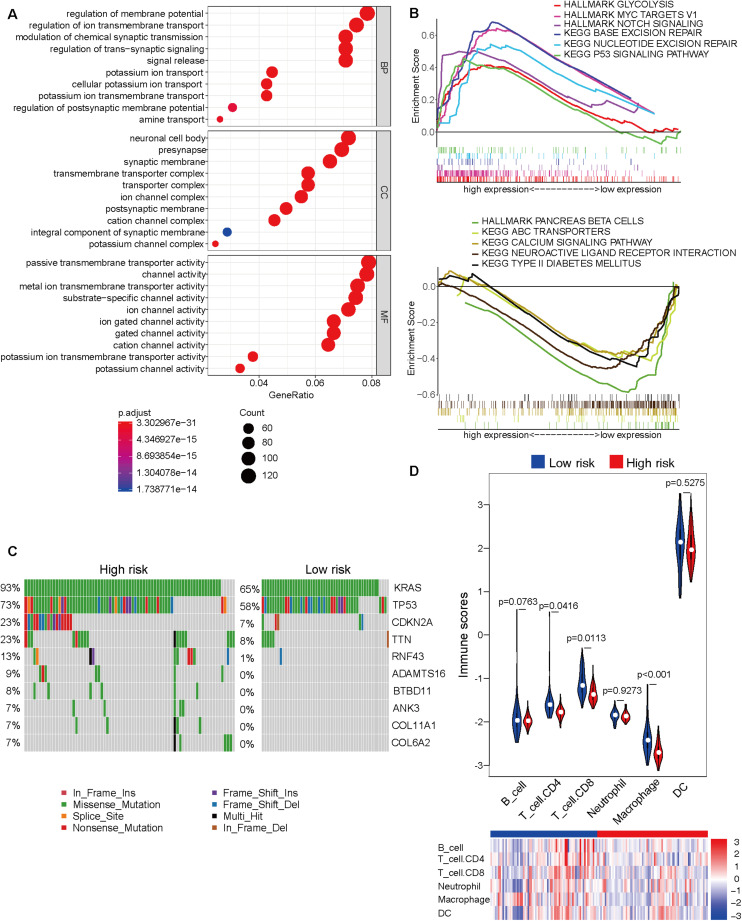
Molecular characteristic analyses of the high-risk and low-risk groups based on the prognostic prediction model. **(A)** The results of Gene Ontology analysis of the high-risk and low-risk groups, including biological process (BP), cellular component (CC), and molecular function (MF). **(B)** The activated (top) and inhibited (bottom) signaling pathways in the high-risk and low-risk groups were subjected to Gene Set Enrichment Analysis (GSEA). **(C)** The comparative mutation spectrum analysis of the top 10 genes in the high-risk and low-risk groups. **(D)** The immune infiltration analysis of six immune cell types in the high-risk and low-risk groups based on the Tumor Immune Estimation Resource (TIMER) website.

The Cancer Genome Atlas dataset was divided into the high-risk and low-risk groups based on the risk score obtained from the prognostic prediction model based on four CpG sites. As the model was significantly associated with the prognosis, it is important to explore the underlying molecular mechanisms. The top 10 results of GO analysis of high-risk and low-risk groups, including molecular function (MF), biological process (BP), and cellular component (CC), are shown in Figure 3A. The GO terms were enriched in several important molecular mechanisms, such as regulation of ion transmembrane transport, regulation of *trans*-synaptic signaling, signal release, presynapse, ion channel complex, postsynaptic membrane, ion channel activity, cation channel activity, and potassium channel activity, which indicated a close relationship between cell signaling transduction and the model. As shown in Figure 3B, the GSEA revealed that glycolysis, MYC targets, Notch signaling, base excision repair, nucleotide excision repair, and p53 signaling pathway were significantly activated, whereas pancreas beta cells, ABC transporters, calcium signaling pathway, neuroactive ligand-receptor interaction, and type II diabetes mellitus were significantly inhibited in the high-risk group. The comparative mutation spectrum analysis identified genes with different mutational frequencies between the high-risk and low-risk groups (Supplementary Table 6). The top 10 genes are shown in Figure 3C. The classical genes associated with the progression of pancreatic cancer, such as *KRAS*, *TP53*, and *CDKN2A* exhibited increased mutational frequency in the high-risk group. Next, the immune cell infiltration was analyzed using the TIMER website. The immune scores of CD4 T cell, CD8 T cell, and macrophage in the high-risk group were significantly lower than those in the low-risk group. This indicated the immunological enhancement of the low-risk group (Figure 3D and Supplementary Table 7).

### Hub Genes Associated With the Prognosis Model Were Identified by WGCNA

**FIGURE 4:**
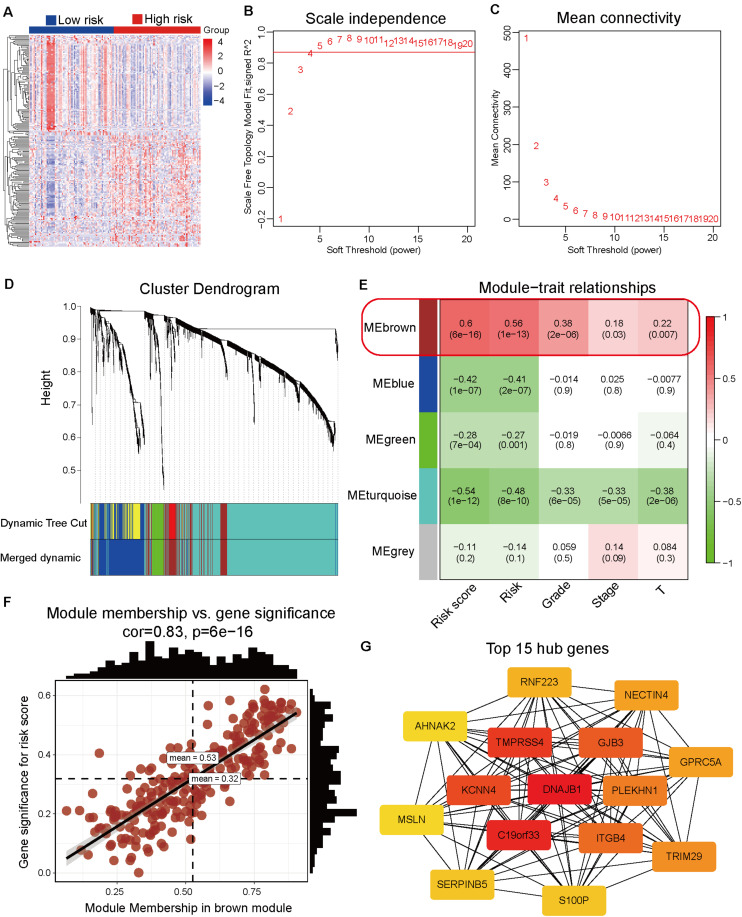
Identification of hub genes associated with the prognosis model by weighted correlation network analysis (WGCNA). **(A)** Heatmap of the differentially expressed genes between the high-risk and low-risk groups. **(B)** The correlation between soft threshold power and scale-free topology model fit. **(C)** The correlation between soft threshold power and mean connectivity. **(D)** Identification of co-expression modules by the hierarchical cluster tree. **(E)** The relationships between gene modules and clinical traits. The correlation coefficient (top) and *p*-value (bottom) of each cell display in the corresponding cell. **(F)** The correlation between module membership and gene significance of the brown module. **(G)** The top 15 hub genes of the brown module are calculated and visualized using the Cytoscape software.

The differentially expressed genes between high-risk and low-risk groups were calculated based on the RNA-Seq data from TCGA dataset. In total, 1861 differentially expressed genes with adjusted *p*-value < 0.05 and | log2 fold-change| > 1 (Supplementary Table 8 and Figure 4A) were obtained. These differentially expressed genes were used as input data for WGCNA to identify the correlations between gene co-expression modules and clinical traits. The best soft power threshold of WGCNA was set as 4 to maintain the scale-free topology and competent connectivity (Figures 4B,C). The hierarchical clustering of WGCNA was utilized to construct five gene co-expression networks (Figure 4D). As shown in Figure 4E, the brown module was significantly correlated with the risk score (correlation coefficient = 0.6, *p* = 6e–16). Moreover, the brown module was significantly positively correlated with tumor grade, clinical stage, and T stage (Figure 4E). These results suggested that the 247 genes in the brown module played a significant role in the progression of pancreatic cancer. The detailed information on the genes of brown module is provided in Supplementary Table 9. To further identify the hub genes of the brown module, the correlation between module membership and gene significance for risk score (Figure 4F) was analyzed. The top 15 hub genes were obtained using the Cytoscape software and *DNAJB1* served as the hub gene of the network (Figure 4G).

### DNAJB1 Was Identified as a Novel Biomarker for Pancreatic Cancer

**FIGURE 5:**
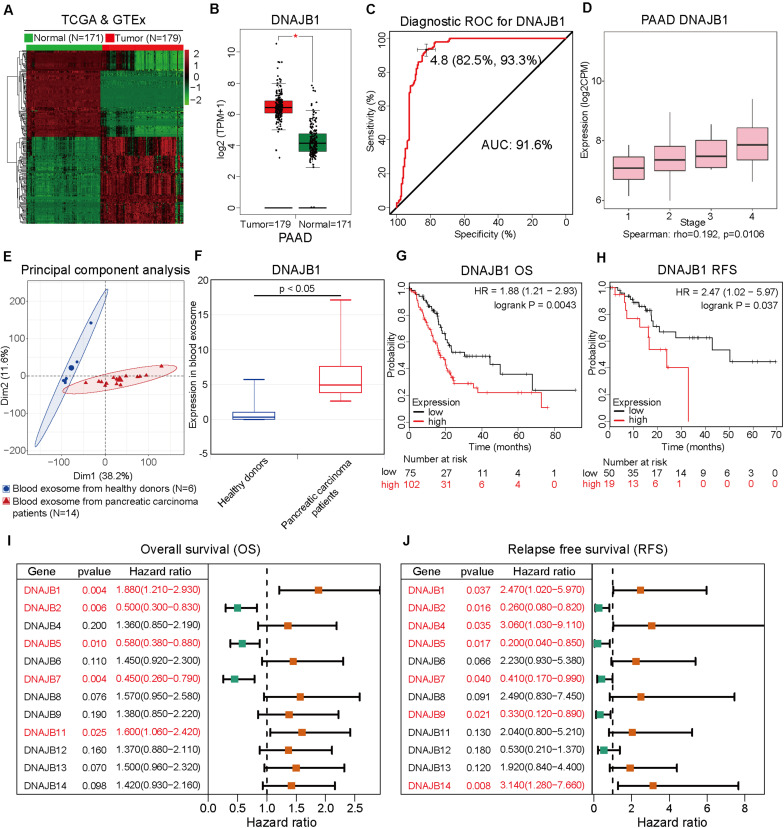
*DNAJB1* serves as a novel biomarker for pancreatic cancer. **(A)** Heatmap of the differentially expressed genes between 171 normal pancreatic tissues and 179 pancreatic cancer tissues based on the integrated analysis of The Cancer Genome Atlas (TCGA) and Genotype-Tissue Expression (GTEx) datasets. **(B)** The relative expression level of *DNAJB1* in 171 normal pancreatic tissues and 179 pancreatic cancer tissues. **(C)** The diagnostic receiving operating characteristic (ROC) curve of *DNAJB1* based on the integrated data from TCGA and GTEx datasets. **(D)** The relationship between *DNAJB1* expression level and different clinical stages of pancreatic cancer. **(E)** The principal component analysis of the blood exosome RNA sequencing (RNA-seq) data of healthy donors (*N* = 6) and patients with pancreatic carcinoma (*N* = 14). **(F)** The relative expression level of *DNAJB1* in the blood exosome of healthy donors (*N* = 6) and patients with pancreatic carcinoma (*N* = 14). **(G)** The overall survival analysis of patients from TCGA dataset based on *DNAJB1* expression. **(H)** The relapse-free survival analysis of patients from TCGA dataset based on *DNAJB1* expression. **(I)** The forest plot shows the overall survival analyses of patients from TCGA dataset based on the expression of *DNAJB* gene family. **(J)** The forest plot demonstrates the relapse-free survival of patients from TCGA dataset based on the expression of *DNAJB* gene family members.

To comprehensively analyze the role of *DNAJB1* and its family members in the progression of pancreatic cancer, a systematic analysis of the *DNAJB* gene family members (*DNAJB1*-*DNAJB9* and *DNAJB11*-*DNAJB14*) was performed. These differentially expressed genes were all obtained from patients with pancreatic cancer based on the stratification of risk score. To verify the diagnostic value of specific genes, the RNA-Seq data of 179 pancreatic cancer tissues and 171 normal pancreatic tissues from TCGA and GTEx databases (Figure 5A) were integrated. As shown in Figure 5B and Supplementary Figure 1A, the expression levels of *DNAJB1*, *DNAJB5*, *DNAJB6*, *DNAJB11*, *DNAJB12*, *DNAJB13*, and *DNAJB14* were significantly upregulated in the tumor tissues. The expression of *DNAJB1* and *DNAJB13* was positively correlated with the clinical stage (Figure 5D and Supplementary Figure 1B, *p* < 0.05). The members of *DNAJB* gene family associated with overall survival and relapse-free survival were analyzed. The detailed information is provided in Figures 5G–J and Supplementary Figure 2. The results indicated that only *DNAJB1* could serve as an unfavorable prognostic factor for overall survival and relapse-free survival. In contrast, *DNAJB2*, *DNAJB5*, and *DNAJB7* served as favorable prognostic factors for overall survival and relapse-free survival. These results demonstrated that *DNAJB1* might serve as a novel biomarker for pancreatic cancer. The diagnostic ROC curve revealed that *DNAJB1* can be used as an effective diagnostic marker, which had a diagnostic value of 4.8 and AUC of 91.6% (95% CI: 82.5–93.3%, Figure 5C). To confirm whether *DNAJB1* can be used as a novel biomarker in the plasma, the plasma exosomal RNA-Seq data of 6 healthy donors and 14 patients with pancreatic carcinoma from the GSE106804 (40) and GSE100232 (41) datasets were downloaded and integrated. The principal component analysis suggested that RNA-Seq data of healthy donors and patients with pancreatic carcinoma clustered separately (Figure 5E). The expression level of exosomal *DNAJB1* was upregulated in patients with pancreatic cancer. A large cohort study is needed to further investigate its diagnostic value (Figure 5F).

**FIGURE 6:**
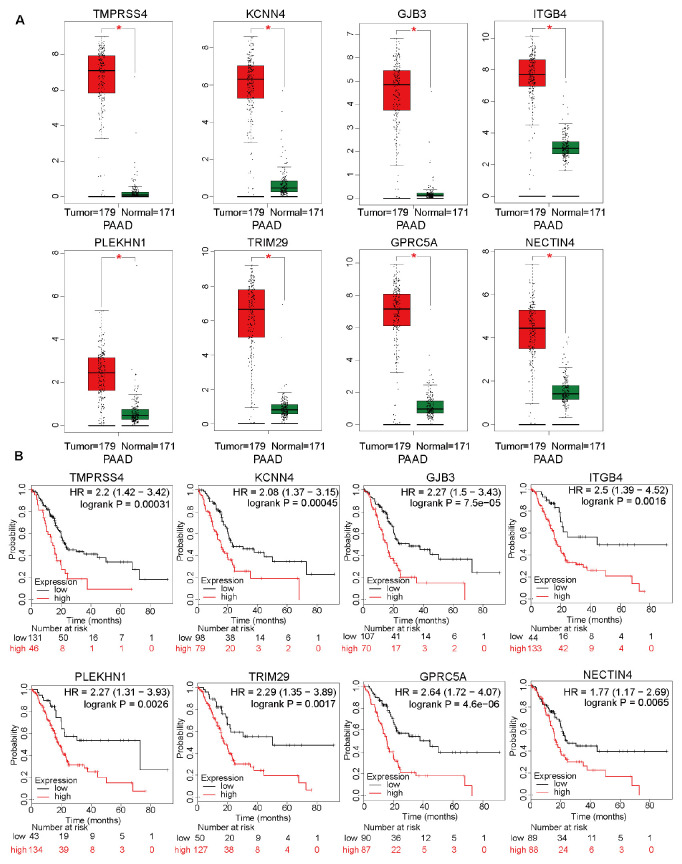
The differential expression levels of top 15 hub genes, except for *DNAJB1*, and analysis of overall survival based on these genes. **(A)** The relative expression levels of *TMPRSS4*, *KCNN4*, *GJB3*, *ITGB4*, *PLEKHN1*, *TRIM29*, *GPRC5A*, and *NECTIN4* in 171 normal pancreatic tissues and 179 pancreatic cancer tissues based on the integrated data from The Cancer Genome Atlas (TCGA) and Genome-Tissue Expression (GTEx) datasets. **(B)** The overall survival analyses of patients from TCGA dataset based on the expression of *TMPRSS4*, *KCNN4*, *GJB3*, *ITGB4*, *PLEKHN1*, *TRIM29*, *GPRC5A*, and *NECTIN4*.

In addition to the top 15 hub genes, the other genes also deserved to be investigated. The expression levels of *TMPRSS4*, *KCNN4*, *GJB3*, *ITGB4*, *PLEKHN1*, *TRIM29*, *GPRC5A*, and *NECTIN4* were also significantly upregulated in the pancreatic cancer tissues (Figure 6A). Moreover, the overall survival analysis indicated that these genes can be used as unfavorable prognostic factors (Figure 6B). These results suggested that WGCNA can effectively select survival-related genes. The detailed roles of these genes in pancreatic cancer should be investigated in future studies.

### DNAJB1 Knockdown Inhibits Malignant Phenotype of Pancreatic Cancer *in vitro* and *in vivo*

**FIGURE 7:**
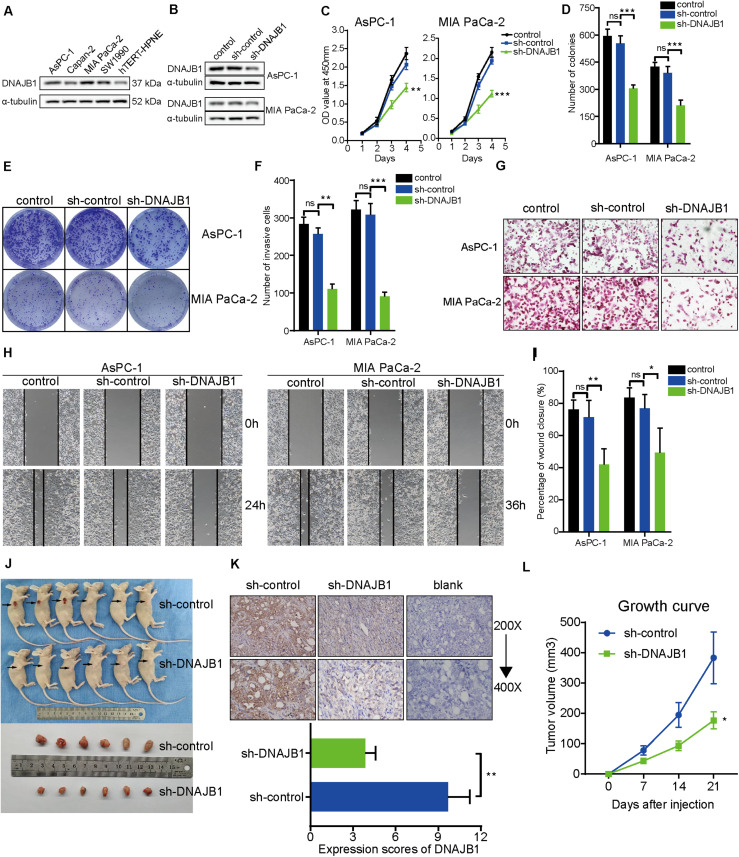
Knockdown of *DNAJB1* inhibits proliferation, migration, and invasion of pancreatic cancer cells *in vitro* and *in vivo*. **(A)** The relative expression level of *DNAJB1* in the AsPC-1, Capan-2, MIA PaCa-2, SW1990, and hTERT-HPNE cell lines detected by western blotting. **(B)** The transfection efficiency of sh-*DNAJB1* in the AsPC-1 and MIA PaCa-2 cell lines detected by western blotting. **(C)** The CCK-8 assay was used to detect the effect of *DNAJB1* knockdown on the proliferation of AsPC-1 and MIA PaCa-2 cell lines. **(D)** Statistical analysis of the colony formation assay results after knockdown of *DNAJB1* in the AsPC-1 and MIA PaCa-2 cell lines. **(E)** Representative images of the colony formation assay, including control, sh-control, and sh-*DNAJB1* groups. **(F)** Statistical analysis of the transwell assay results after knockdown of *DNAJB1* in the AsPC-1 and MIA PaCa-2 cell lines. **(G)** Representative images of the transwell assay. **(H)** Representative images of the wound healing assay. **(I)** Statistical analysis of the wound healing assay results after knockdown of *DNAJB1*. **(J)** Subcutaneous tumor tissues of sh-control and sh-*DNAJB1* groups at 3 weeks after initial implantation. **(K)** Relative *DNAJB1* expression in the tumor tissues excised from sh-control and sh-*DNAJB1* groups was detected by immunohistochemical assay. **(L)** The growth curve of subcutaneous tumor tissues of sh-control and sh-*DNAJB1* groups. *p < 0.05, **p < 0.01, and ***p < 0.001.

To evaluate the specific role of *DNAJB1* in pancreatic cancer, the relative expression level of *DNAJB1* in the four pancreatic cancer cell lines (AsPC-1, Capan-2, MIA PaCa-2, and SW1990) and a hTERT-HPNE was analyzed. The expression level of *DNAJB1* in the AsPC-1 and MIA PaCa-2 cell lines was higher than that in the other cell lines (Figure 7A). Therefore, the AsPC-1 and MIA PaCa-2 cell lines were chosen for further functional assays. The efficiency of *DNAJB1* knockdown was detected by western blotting (Figure 7B). The results of CCK8 and colony formation assays indicated that *DNAJB1* knockdown significantly inhibited the proliferation and colony formation rate of AsPC-1 and MIA PaCa-2 cells (Figures 7C–E). The results of transwell assay revealed that the AsPC-1 and MIA PaCa-2 cells exhibited markedly decreased invasion upon *DNAJB1* knockdown (Figures 7F,G). The results of wound healing assay demonstrated that the knockdown of *DNAJB1* significantly decreased the migration of AsPC-1 and MIA PaCa-2 cells (Figures 7H,I). The subcutaneous xenograft model was utilized to detect the cellular proliferation ability *in vivo*. The group injected with *DNAJB1* knockdown AsPC-1 cells exhibited significantly smaller tumoral volumes than the negative control group (Figures 7J,K). The relative expression of *DNAJB1* was detected by the immunohistochemical assay (Figure 7L). These results indicated that *DNAJB1* may be a novel promoter of pancreatic cancer. Further studies are needed to elucidate the underlying molecular mechanisms.

## Discussion

Pancreatic cancer, a malignancy associated with high mortality, has high heterogeneity. The patients with pancreatic cancer receiving similar therapies exhibit varied clinical outcomes (42). Therefore, the development of a risk stratification model may help clinicians to design personalized treatment programs for different patients. Previous studies have proposed various risk stratification models for the diagnosis, prognosis, and recurrence of pancreatic cancer, which exhibited better efficiency than the classical TNM stage (43–45). The rapid development of sequencing techniques has enabled the access to multi-omics data and high-quality clinical information through different databases, such as TCGA, ICGC, and Gene Expression Omnibus (17, 46, 47). These resources provide novel insights into the initiation and progression of multiple cancers. There is an urgent need to devise strategies to increase the overall survival of patients with pancreatic cancer. The development of a prognosis prediction model based on genome data can be a useful tool for molecular and precise medicine.

Several recent studies have demonstrated that the prognosis model based on DNA methylation data can be used to predict the prognosis of patients with various cancers with satisfactory efficiency. The circulating tumor DNA methylation markers can be utilized to generate a risk model for the diagnosis and prognosis prediction of ovarian cancer, colorectal cancer, and hepatocellular carcinoma (13, 48, 49). Additionally, the DNA methylation markers originating from tissues can also be employed to construct a prognosis prediction model for esophageal, gastric, and prostate cancers (11, 12, 14). These studies demonstrated that the DNA methylation status can serve as novel biomarkers to generate the prognosis prediction model. However, there are limited studies that have reported the significance of prognosis prediction model in pancreatic cancer. In this study, a prognostic prediction model based on four CpG sites, namely cg00609645, cg13512069, cg23811464, and cg03502002, was established. The model was generated based on TCGA dataset and the conclusion was verified in the external ICGC dataset. Next, a nomogram model was constructed based on the independent prognostic factors of pancreatic cancer. Chen H, et al. used three hypomethylated genes (SULT1E1, IGF2BP3, and MAP4K4) to construct a prognostic prediction model using the AUC (1-year: 0.62, 3-year: 0.69, and 5-year: 0.69) (50). Liao X, et al. had constructed a prognostic model comprising 9 hub genes and reported that the AUC for 1-, 3-, and 5-year was 0.641, 0.623, and 0.554, respectively (51). Compared to these two models, the nomogram model exhibited better prediction ability using the AUC (1-year: 0.81, 3-year: 0.91, and 5-year: 0.89). These results suggested that the nomogram model can be employed as an effective instrument for prognosis prediction in patients with pancreatic cancer. The model can be improved with increased access to sequencing data and clinical information.

To further elucidate the molecular mechanisms underlying the prognostic model, the GO, GSEA, mutation spectrum, and immune infiltration analyses were performed on the subgroups stratified by the prognostic prediction model. The low-risk group with improved prognosis exhibited less mutational frequency and high immune cell infiltration. The analysis of several important signaling pathways in the subgroups can aid in a better molecular understanding of the prognostic model.

Furthermore, the WGCNA was performed using the clinical traits and differentially expressed genes. The brown module containing 247 genes was significantly correlated with the prognostic model, tumor grade, clinical stage, and T stage. Next, *DNAJB1* was identified as the hub gene of the brown gene module. These results indicated that *DNAJB1* can play a vital role in pancreatic cancer. Previous studies have reported that *DNAJB1* expression, which is upregulated in the tissues, cell lines, and bile of cholangiocarcinoma, can serve as a new biomarker for cholangiocarcinoma (52). *DNAJB1*-*PRKACA* gene fusion is reported to play an oncogenic promoter role in fibrolamellar hepatocellular carcinoma (53, 54). In addition, several researches have demonstrated that the *DNAJB1–PRKACA* gene fusion can also be found in the pancreatic and biliary intraductal oncocytic papillary neoplasm (IOPN), as well as in the intraductal papillary mucinous neoplasm (IPMN) of pancreas and pancreatic ductal adenocarcinoma. The specific functions of the gene fusion in the initiation and progression of IOPNs, IPMNs, and their associated neoplasms need further research (55, 56). Cui X, et al. reported that *DNAJB1* can suppress apoptosis and promote cancer cell proliferation via ubiquitin degradation of PDCD5 in the lung cancer cell line (A549) (57). To identify the specific role of *DNAJB1* in pancreatic cancer, a systematic analysis of DNAJB family members was performed. The analysis indicated that *DNAJB1* may serve as a novel biomarker for the diagnosis and prognosis of pancreatic cancer. The role of *DNAJB1* in the proliferation, migration, and invasion of pancreatic cancer cells was verified *in vivo* and *in vitro*. The molecular mechanisms of *DNAJB1* in pancreatic cancer must be elucidated in future studies.

## Conclusion

A novel prognostic prediction model was established based on four CpG sites for pancreatic cancer. The molecular characteristic analyses based on the model provided new insights into the initiation and development of pancreatic cancer. The WGCNA can serve as an excellent tool to identify the genes correlated with specific clinical traits. *DNAJB1* can serve as a potential diagnostic and prognostic biomarker for pancreatic cancer.

## Data Availability Statement

All datasets presented in this study are included in the article/Supplementary Material.

## Ethics Statement

The animal study was reviewed and approved by the Ethics Committee of Shengjing Hospital of China Medical University.

## Author Contributions

LK, PL, and XT contributed to conception and design of the study. XF, TW, and ZW organized the database. LK and PL performed the statistical analysis. LK and PL wrote the first draft of the manuscript. LK, PL, BZ, and JL wrote sections of the manuscript. All authors contributed to manuscript revision, read, and approved the submitted version.

## Conflict of Interest

The authors declare that the research was conducted in the absence of any commercial or financial relationships that could be construed as a potential conflict of interest.

## Footnotes

1 https://portal.gdc.cancer.gov/
