# Supplementary material for: A Prognostic Prediction Model Developed Based on Four CpG Sites and Weighted Correlation Network Analysis Identified DNAJB1 as a Novel Biomarker for Pancreatic Cancer
Source: Front Oncol. 2020 Aug 25;10:1716. doi: 10.3389/fonc.2020.01716 (PMC7477361; doi:10.3389/fonc.2020.01716)
Supplement: Supplementary file 1 [file Data_Sheet_1.PDF]

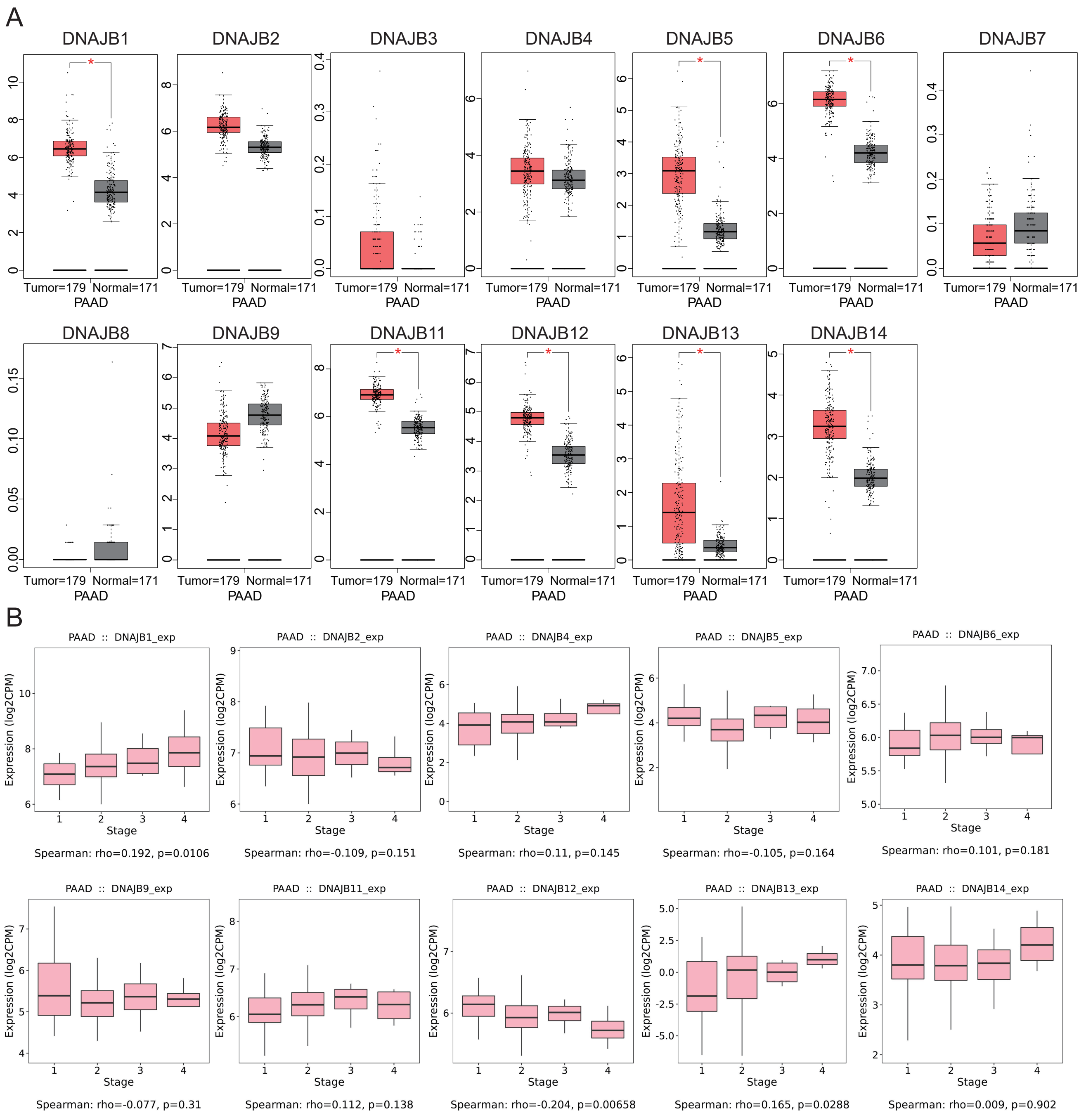

Supplementary Figure 1. The differential expression levels and correlations with the clinical stages of DNAJB gene family members. (A) Relative expression level of DNAJB gene family members in 171 normal pancreatic tissues and 179 pancreatic cancer tissues. (B) The correlation of the expression level of DNAJB gene family members and clinical stages.

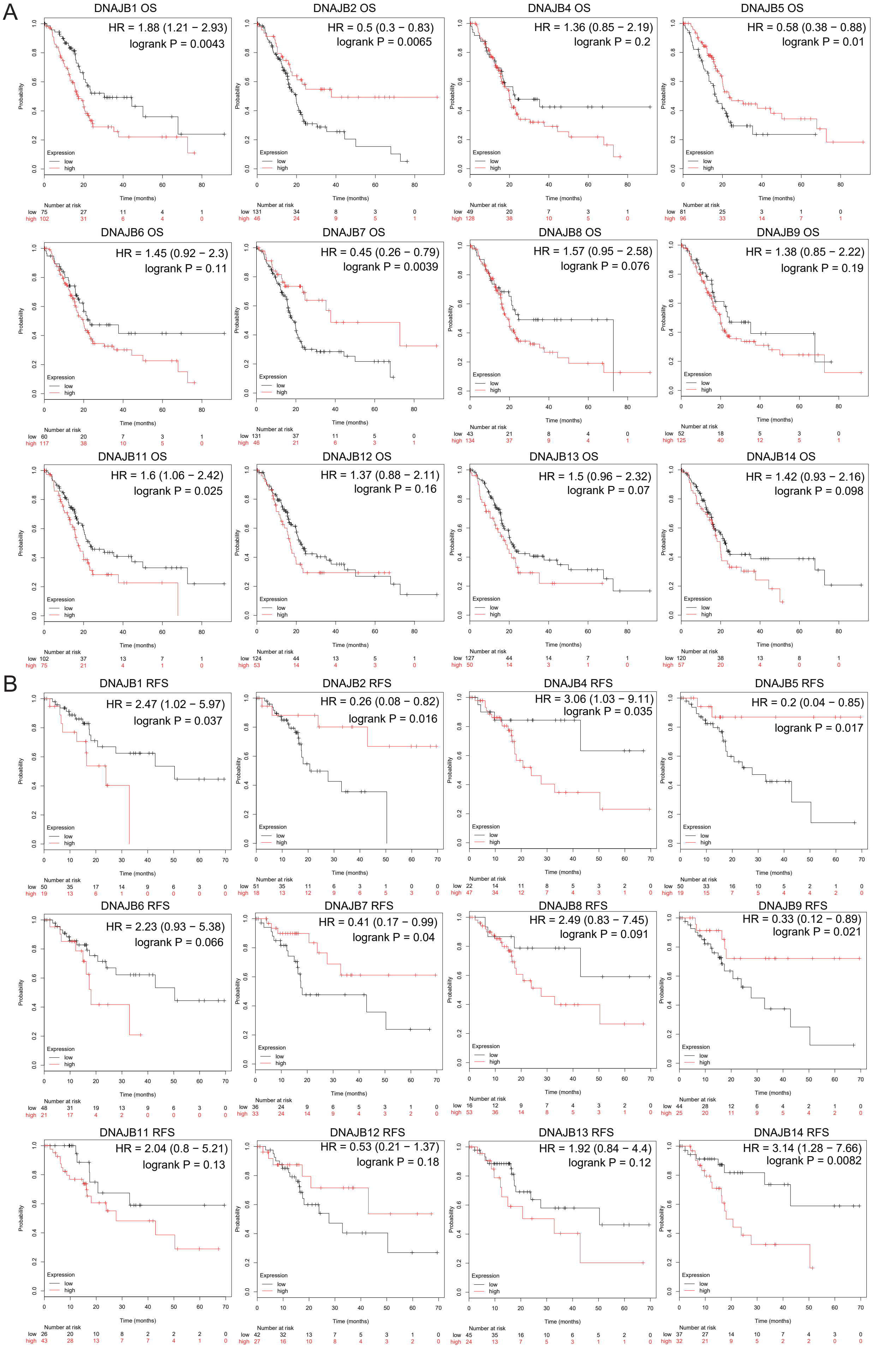

Supplementary Figure 2. The overall survival and relapse-free survival analyses of patients based on the expression level of DNAJB gene family members. (A) The overall survival analyses of patients based on the expression level of DNAJB gene family members. (B) The relapse-free survival analyses of patients based on the expression level of DNAJB gene family members.
